# Exploring the Physiological Multiplicity of Native Microalgae from the Ecuadorian Highland, Italian Lowland and Indoor Locations in Response to UV-B

**DOI:** 10.3390/ijms24021346

**Published:** 2023-01-10

**Authors:** Thais Huarancca Reyes, Carolina Chiellini, Emilio Barozzi, Carla Sandoval, Cristina Echeverría, Lorenzo Guglielminetti

**Affiliations:** 1 Department of Agriculture, Food and Environment, University of Pisa, Via Mariscoglio 34, 56124 Pisa, Italy; 2 Institute of Agricultural Biology and Biotechnology, Italian National Research Council, Via Moruzzi 1, 56124 Pisa, Italy; 3 Department of Biotechnology, Universidad Técnica del Norte, Av. 17 de Julio 5-21 y Gral. José María Córdova, Ibarra 100150, Ecuador

**Keywords:** chlorophyll fluorescence, microalgae, phenolic compounds, photosynthetic pigments, non-enzymatic antioxidants, ultraviolet light

## Abstract

The differential effects of UV-B on the inhibition or activation of protective mechanisms to maintain cells photosynthetically active were investigated in native microalgae. Four strains were used, including two *Chlorella sorokiniana* strains, F4 and LG1, isolated from a Mediterranean inland swamp and a recycled cigarette butt’s substrate, respectively, and two isolates from an Ecuadorian highland lake related to *Pectinodesmus pectinatus* (PEC) and *Ettlia pseudoalveolaris* (ETI). Monocultures were exposed to acute UV-B (1.7 W m^−2^) over 18 h under controlled conditions. UV-B-untreated microalgae were used as the control. Comparative physiological responses, including photosynthetic pigments, non-enzymatic antioxidants, and chlorophyll *a* fluorescence, were evaluated at specific time points. Results showed that UV-B significantly compromised all the physiological parameters in F4, thereby resulting in the most UV-B-sensitive strain. Contrarily, UV-B exposure did not lead to changes in the PEC physiological traits, resulting in the best UV-B-resistant strain. This could be attributed to the acclimation to high light habitat, where maintaining a constitutive phenotype (at the photosynthetic level) is strategically advantageous. Differently, LG1 and ETI at 12 h of UV-B exposure showed different UV-B responses, which is probably related to acclimation, where in LG1, the pigments were recovered, and the antioxidants were still functioning, while in ETI, the accumulation of pigments and antioxidants was increased to avoid further photodamage. Consequently, the prolonged exposure in LG1 and ETI resulted in species-specific metabolic regulation (e.g., non-enzymatic antioxidants) in order to constrain full photoinhibition under acute UV-B.

## 1. Introduction

Microalgae are an extremely diverse group of mostly photosynthetic microorganisms that inhabit aquatic and terrestrial environments. As photoautotrophic organisms, they use sunlight as an energy source, specifically the visible light spectrum known as photosynthetically active radiation (PAR) (400–700 nm) [1]. In addition to PAR, the Sun also irradiates ultraviolet (UV) light, which is divided into low-energy UV-A (315–400 nm), and high-energy UV-B (280–315 nm) and UV-C (100–280 nm) radiation [2,3]. Years ago, only radiation with wavelengths greater than 290 nm reached Earth’s surface [4]. Today, due to the depletion of the ozone layer, the presence of UV-B radiation is a world concern [5]. This type of radiation causes cell damage in almost all living organisms, including microalgae, as UV-B can also penetrate the water column to a considerable depth [6,7,8].

In microalgae, UV-B can overstimulate photosynthesis, inducing the formation of reactive oxygen species (ROS), which in turn changes lipid and fatty acid structures, DNA components, and chloroplast functionality [9,10]. Moreover, the increasing exposure to solar radiation over time has forced some species to develop gradual adaptation mechanisms against light stress. These mechanisms include photo-repair, antioxidant system activation, and biosynthesis of photoprotective compounds, such as mycosporine-like amino acids (MAAs), scytonemin (Scy), carotenoids, and polyamines [11,12,13]. Recently, the UV-B photoreceptor, UVR8 (UV RESISTANCE LOCUS 8), which was first described in the model plant *Arabidopsis thaliana*, was identified in angiosperms, lycophytes, bryophytes, and green algae [14,15,16]. Subsequently, Zhang et al. [17] found that the mechanism of action of the core UVR8 signaling pathway is well conserved in green algae. 

Most of the studies have been focused on the use of UV-B as a tool in the microalgal machinery of biorefineries in order to stimulate the biosynthesis of valuable biomolecules [18,19]. However, less is known about the detailed defense mechanisms involved in the UV-B response in microalgae, especially in those native strains adapted to extreme and unexplored ecosystems. For instance, the high Andean lakes are a still unexplored source of photosynthetic microorganisms that are well adapted to extreme environmental conditions characteristic of the equatorial zone, such as altitude, high UV radiation, constant temperatures, and constant photoperiods [20]. Therefore, the aim of the present study was to investigate the differential effects of UV-B on the inhibition or activation of protective mechanisms in native microalgae from highland, lowland, and indoor conditions under an eco-physiological context.

In the attempts to elucidate the mechanisms maintaining the microalgal cells’ photosynthetic activity in response to UV-B, the production of photosynthetic pigments (chlorophyll *a*, chlorophyll *b*, and carotenoids), total antioxidant capacity, total polyphenols, and chlorophyll *a* fluorescence were evaluated in four strains exposed to acute UV-B irradiation and compared with their control (absence of UV-B). The strains were isolated from different sources: two from the Ecuadorian Yahuarcocha Lake located at 2200 m asl (00°22′10″ N; 78°06′09″ W), one from a recycled cigarette butt’s substrate in a growth chamber [21], and the last one from the Mediterranean Fucecchio marshland at sea level (43°48′31″ N; 10°48′18″ E) [22]. Herein, we discuss the distinct ability of each native microalgal strain to cope with acute UV-B, which was related to their respective natural environment and genetic constitutions.

## 2. Results

### 2.1. Identification of the Strains PEC and ETI

The Yahuarcocha Lake is mainly inhabited by metaphytic and benthic communities such as diatoms, chlorophyceans, and cyanobacteria [23]. In this study, the microalgal strains PEC and ETI were identified as *Pectinodesmus pectinatus* and *Ettlia pseudoalveolaris*, respectively. The comparisons of amplified sequences base on GeneBank are described in Table 1.

### 2.2. Effects of UV-B on the Photosynthetic Pigments of Microalgal Strains

**Figure 1:**
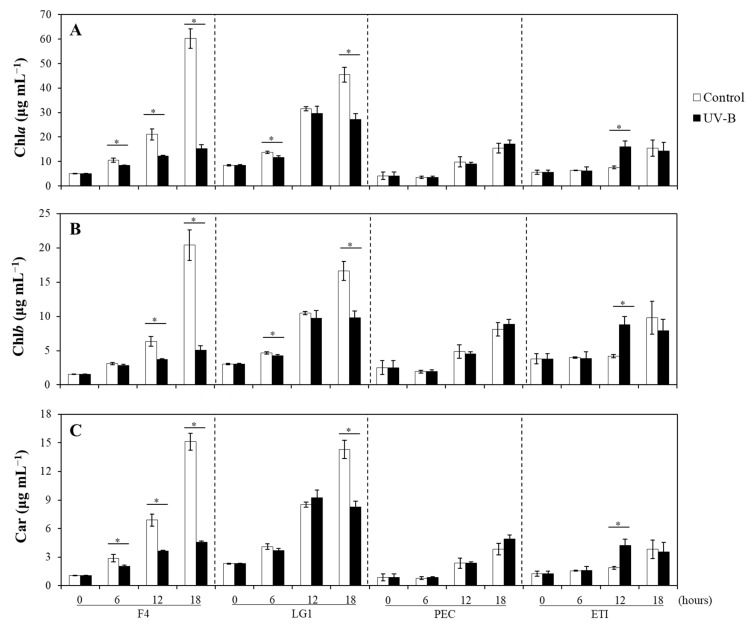
Effects of UV-B on the photosynthetic pigments. (**A**) Chlorophyll *a* (Chl*a*), (**B**) chlorophyll *b* (Chl*b*), and (**C**) carotenoids (Car) concentrations were determined in control (bars in white) and UV-B treated (bars in black) microalgal strains (F4, LG1, PEC, and ETI). UV-B exposure level was set at 1.7 W m^−2^ supplemented with 70 μmol m^−2^ s^−1^ photosynthetically active radiation (PAR) following a time course from 0 to 18 h. Control microalgal group only received PAR. Asterisks represent significant differences (* *p* < 0.05) between control and UV-B treatment within the same strain at a specific time point. Data are expressed as means of 3 different replicates ± standard deviation (SD). F4 and LG1: *Chlorella sorokiniana* strains; PEC: *Pectinodesmus pectinatus* strain; ETI: *Ettlia pseudoalveolaris* strain.

In general, all photosynthetic pigments increased over time, with some differences between control and UV-B-treated microalgae depending on the species (Figure 1). Among *Chlorella sorokiniana* strains, F4 exposed to UV-B showed significantly lower content of chlorophyll *a* (Chl*a*) than those cells under control conditions, while Chl*a* in LG1 only were significantly lower when cells were exposed for 6 and 18 h UV-B with respect to their controls (Figure 1A). Differently, Chl*a* in the Andean microalgal species did not show differences between control and UV-B treated cells over time except for ETI at 12 h treatment, where UV-B treated cells showed significantly higher levels of Chl*a* than control cells (Figure 1A). The content of Chl*b* in all species showed similar patterns to Chl*a* except for F4 at 6 h treatment, which did not show differences between control and UV-B treated cells (Figure 1B). Carotenoids (Car) in all species showed similar patterns to Chl*a* except for LG1 at 6 h treatment, which did not show differences between control and UV-B treated cells (Figure 1C).

### 2.3. Effects of UV-B on the Non-Enzymatic Antioxidants of Microalgal Strains

**Figure 2:**
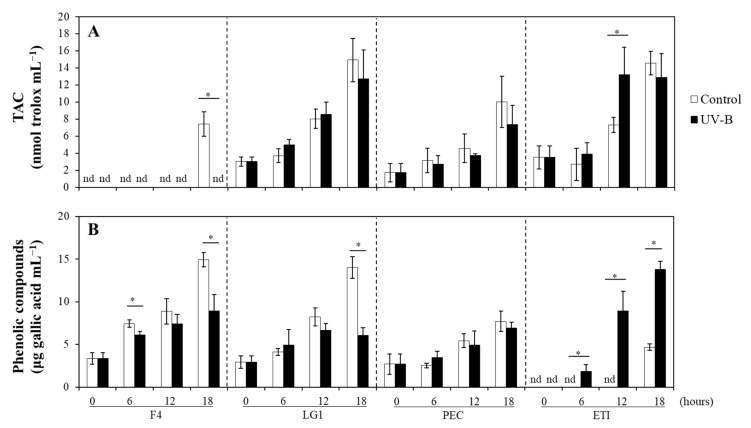
Effects of UV-B on the non-enzymatic antioxidants. (**A**) Total antioxidant capacity (TAC) and (**B**) phenolic compounds were determined in control (bars in white) and UV-B treated (bars in black) microalgal strains (F4, LG1, PEC, and ETI). UV-B exposure level was set at 1.7 W m^−2^ supplemented with 70 μmol m^−2^ s^−1^ photosynthetically active radiation (PAR) following a time course from 0 to 18 h. Control microalgal group only received PAR. Asterisks represent significant differences (* *p* < 0.05) between control and UV-B treatment within the same strain at a specific time point. Data are expressed as means of 3 different replicates ± standard deviation (SD). nd: not detectable. F4 and LG1: *Chlorella sorokiniana* strains; PEC: *Pectinodesmus pectinatus* strain; ETI: *Ettlia pseudoalveolaris* strain.

The total antioxidant capacity (TAC) in LG1, PEC, and ETI generally increased over time without differences between control and UV-B treated microalgae except for ETI at 12 h treatment, where significant differences were observed between control and UV-B (Figure 2A). TAC in F4 was not detectable over time, neither in control nor UV-B treated cells, with the exception at 18 h treatment under control conditions, when TAC was detected (Figure 2A). At control conditions, the phenolic compounds in F4 and LG1 gradually increased over time; however, these patterns were affected upon UV-B irradiation at specific time points (Figure 2B). In detail, F4 only showed significant differences between control and UV-B treated cells at 6 and 18 h, while LG1 showed differences at 18 h of treatment, where a decrease in phenolic compounds was observed due to UV-B (Figure 2B). Concerning the Andean microalgal species, PEC showed a gradual increase in phenolic compounds over time, and significant differences were not detected between control and UV-B treated cells (Figure 2B). The phenolic compounds in control ETI were detectable only at the end of the experiment (i.e., 18 h), while in UV-B treated cells started to be detectable beyond 6 h following a gradual increase over time with significantly higher values than their respective control (Figure 2B).

### 2.4. Effects of UV-B on the Chlorophyll a Fluorescence of Microalgal Strains

**Figure 3:**
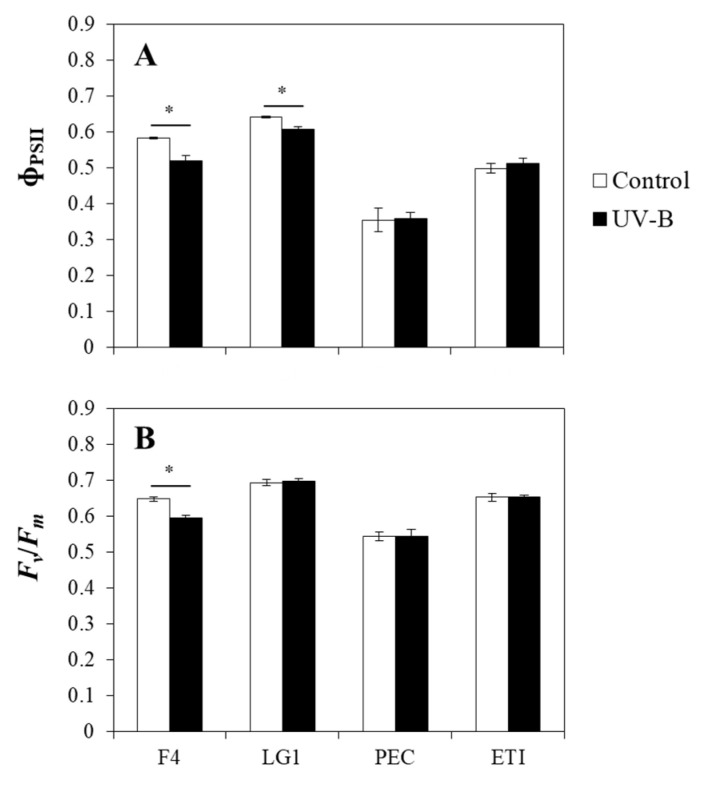
Effects of UV-B on the chlorophyll *a* fluorescence. The (**A**) actual (Φ_PSII_) and (**B**) maximum (*F_v_*/*F_m_*) efficiency of photosystem II (PSII) photochemistry were determined in control (bars in white) and UV-B treated (bars in black) microalgal strains (F4, LG1, PEC, and ETI). Measurements were performed at 18 h of treatment. UV-B exposure level was set at 1.7 W m^−2^ supplemented with 70 μmol m^−2^ s^−1^ photosynthetically active radiation (PAR). Control microalgal group only received PAR. Asterisks represent significant differences (* *p* < 0.05) between control and UV-B treatment within the same strain. Data are expressed as means of 6 different replicates ± standard deviation (SD). F4 and LG1: *Chlorella sorokiniana* strains; PEC: *Pectinodesmus pectinatus* strain; ETI: *Ettlia pseudoalveolaris* strain.

The UV-B treated F4 and LG1 showed significantly lower values of the actual (Φ_PSII_) efficiency of PSII photochemistry in comparison with their control cells; however, PEC and ETI did not show significant differences in their Φ_PSII_ values when control and UV-B treatment were compared (Figure 3A). Concerning the results of the maximum (*F_v_*/*F_m_*) efficiency of PSII photochemistry, F4 was the only microalgal strain that showed significant differences between control and UV-B treated cells, where UV-B caused a decrease in *F_v_*/*F_m_* (Figure 3B).

### 2.5. Multiple Factor Analysis

**Figure 4:**
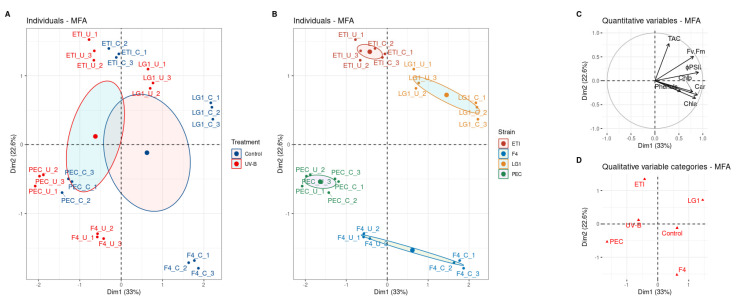
Multiple factor analysis (MFA) of physiological data in microalgae. (**A**,**B**) Score plots of the two-first principal components describing the strains and treatments. Representation of (**C**) groups of quantitative variables, and (**D**) categories of qualitative variables. Control and UV-B irradiated microalgal strains (F4, LG1, PEC and ETI) at the end of the treatment. F4 and LG1: *Chlorella sorokiniana* strains; PEC: *Pectinodesmus pectinatus* strain; ETI: *Ettlia pseudoalveolaris* strain; Control: non-treated microalgal strains; UV-B: microalgal strains exposed to UV-B; TAC: total antioxidant capacity; Fv.Fm: the potential efficiency of photosystem II (PSII) photochemistry represented by the ratio *F_v_*/*F_m_*; ΦPSII: the photon yield of PSII photochemistry in the light; Chla: chlorophyll *a*; Chlb: chlorophyll *b*; Car: carotenoids. In this analysis, values indicated with nd (not detectable) were approximated with “0” values.

The multiple factor analysis (MFA) showed that on the first dimension (33% of the explained variability), the microalgal strains were divided on the base of the treatment (Figure 4A,D), confirming an effect of the exposure to UV-B in all the tested microalgal strains. Accordingly, replicates of controls and treatments were separated in each microalgal strain (Figure 4A). On the second dimension (22.6% of the variability, y-axis), the separation between two distinct groups of microalgal strains occurred (Figure 4B), defining physiological differences between the group PEC and F4 (downside of the plot), with ETI and LG1 (upper side of the plot). Considering the quantitative variable “TAC” (Figure 4C), the TAC variable vector in the ordination space was almost parallel with the second component, suggesting its high contribution to the component; accordingly, it differentiated very well between these two groups of algae, showing greater values in the ETI and LG1 groups. According to the vectors of the quantitative variables (Figure 4C), strain LG1 exposed to UV-B treatment showed greater photosynthetic performance both in the presence and absence of UV-B treatment, with respect to the other strains. On the other side, strain PEC, both treated and not treated, as well as strain F4 exposed to UV-B, showed lower values of TAC, *F_v_*/*F_m_*, and Φ_PSII_ (Figure 4C).

## 3. Discussion

Photosynthesis is one of the physiological processes that are a main target of UV-B radiation. Although some microalgae subjected to enhanced UV-B resulted in a decline in photosynthetic pigments and yield [26], others are also able to acclimate to UV-B by adjusting their metabolism and preventing damage [16,27,28]. In this study, the pigment content in *Chlorella sorokiniana* strains was differently inhibited by the continuous UV-B, where F4 was unexpectedly more susceptible than LG1 despite only the former having naturally grown in the presence of ambient UV-B [21,22]. In fact, the native habitat of strain F4 was a Mediterranean inland swamp at sea level with an average depth of 2 m and was historically affected by anthropogenic activities [29]. Living organisms grown in outdoor environments are simultaneously exposed to diverse stresses triggering a complex interaction between signaling pathways [30]. Thus, it is possible that in F4, the applied acute UV-B resulted in a negative stress due to the lack of other co-occurring stresses, as demonstrated in a recent study where the UV-B acted as a ‘positive stress’ when it was presented with another abiotic variable [31]. Consistently, F4 exposed to continuous acute UV-B (1.7 W m^−2^) for 18 h resulted in a significant decline of the photosynthetic activity, indicating that this acute UV-B dose induced photoinhibition probably due to the imbalance of pro-oxidant production and scavenging capacity in F4 cells [32,33,34,35].

Unlike F4, LG1 was acclimated to only PAR light and controlled indoor conditions [21]. In this study, when LG1 was exposed to acute UV-B for 6 h, it caused a transient reduction in chlorophyll content that was then recovered to control levels when exposure increased to 12 h. This indicated a possible acclimation process in LG1 cells where damage and repair were rebalancing to maintain their physiological performance [36,37]. Interestingly, the content of carotenoids and non-enzymatic antioxidants as phenolic compounds were maintained at control levels when LG1 was exposed to up to 12 h UV-B, indicating that these antioxidants were still functioning in this treatment period and played an important photoprotective role during UV-B acclimation [13,38]. Conversely, increasing the UV-B exposure to 18 h resulted in inhibitory effects on photosynthetic pigment content; however, these levels were similar to those in cells exposed to 12 h UV-B. It has been reported that UV-B may have a harmful impact on photosynthetic pigments due to their photobleaching or biosynthesis inhibition and, thus, leading to a decrease in photosynthetic performance [13,39]. On the other hand, UV-B-induced photoinhibition, reflected in the decrease in *F_v_*/*F_m_*, may be ameliorated by prior UV-B acclimation [16]. In fact, microalgae are able to adapt the number and size of their photosynthetic units when exposed to prolonged high irradiance; thus, the photoacclimation process may include the downregulation of pigment content in order to prevent photodamage [36,40]. In this study, *F_v_*/*F_m_* retained its maximum value while Φ_PSII_ was reduced in 18 h UV-B-exposed LG1 cells, indicating that the photosynthetic processes in LG1 are resistant to acute UV-B doses as UV-B-induced decline in Φ_PSII_ did not lead to full inhibition. Such resistance of LG1 against acute UV-B may be attributed to the effective energy dissipation via regulated and non-regulated non-photochemical pathways and the involvement of various metabolites in UV-B acclimation response [41,42]. Indeed, TAC content was maintained at control levels during the experimental period, indicating that these non-enzymatic antioxidants were still functioning and were important during UV-B acclimation. Since enzymatic and non-enzymatic antioxidants may have a good complementary function in the protection of cells from UV-induced photo-oxidative damage [9,32,35], it would be interesting to identify those metabolites involved in LG1 photoacclimation.

Regarding the highland microalgal strains (i.e., PEC and ETI), their photosynthetic pigments and efficiency were markedly lower than in *Chlorella* strains under control conditions, which might be related to the adaptation mechanisms to the rough climate conditions characterized by high solar radiation. Thus, it is plausible that to cope with too much light energy in the Ecuadorian highland, the strains PEC and ETI have strategically altered the size of chlorophyll antenna or the number of light-harvesting complexes in order to avoid photoinhibition but maintain their cells’ photosynthetic activity [43]. Although the strains PEC and ETI were isolated from the same habitat, they showed different physiological responses to continuous acute UV-B radiation, especially those related to the antioxidant systems, suggesting that their abilities to cope with UV-B might be based on their genetic constitutions. In detail, the UV-B exposed cells of PEC maintained their content of chlorophylls and carotenoids at control levels over the time course, indicating that this strain was quite resistant to acute UV-B radiation. Such resistance may be attributed to the evolutionary capacity to maintain a reduced number and size of photosynthetic units, and thus, protecting its photosynthetic machinery from photodamage. Concordantly, PEC showed the lowest photosynthetic activity in comparison with the other strains. Moreover, the fact that the content of non-enzymatic antioxidants as phenolic compounds was also maintained at control levels in UV-B exposed cells indicates that the pro-oxidant production and scavenging capacity were balanced in PEC, even under acute UV-B. However, it would be interesting to evaluate the involvement of other types of antioxidants (e.g., enzymatic scavengers) in response to UV-B [12,13,26,27,32].

Although the highland microalgal strains did not show negative effects on their photosynthetic performances in response to acute UV-B, the *Ettlia pseudoalveolaris* strain ETI displayed generally higher photosynthetic activity than the *Pectinodesmus pectinatus* PEC. This indicates that features of the UV-B acclimation differ between species, probably due to their genetic constitutions. It has been demonstrated that the UV-B photoreceptor UV RESISTANT LOCUS8 (UVR8) signaling pathway originated in chlorophytes and facilitated green algae to adapt to shallow water environments [17,44]. More in detail, recent studies in *Chlamydomonas reinhardtii*, from the order Chlamydomonales as ETI, showed that UV-B acclimation mediated by UVR8 activation can prevent UV-B-induced photoinhibition by the alteration of the repair rate of PSII-related proteins, induction of specific genes encoding chloroplast proteins related to nonphotochemical quenching, and accumulation of xanthophyll pigments [16,45,46]. In this study, ETI showed a marked induction of chlorophylls, carotenoids, and non-enzymatic antioxidants as phenolic compounds upon 12 h UV-B. Concordantly, the accumulation of chlorophyll in *Dunaliella salina* (Chlamydomonales) in response to enhanced UV-B was related to acclimation mechanisms regulated by specific photoreceptors [27,47]. Moreover, it is well known that successful acclimation to UV-B consists of maintaining pro-oxidants and antioxidants in balance [32,48]. For instance, in microalgae, the biosynthesis and accumulation of non-enzymatic antioxidants, such as carotenoids and phenolic compounds, are common strategies to cope with the detrimental UV-B effects and maintain cellular integrity [13]. Altogether, it indicates that 12 h of acute UV-B exposure in ETI allowed UV-B acclimation response, including increased accumulation of phenolic compounds and carotenoids that reduce photodamage to the photosynthetic machinery. In fact, the prolonged UV-B exposure (i.e., 18 h) did not affect the photosynthetic performance of this strain, probably due to its precedent acclimation. Further studies are needed to reveal whether UVR8 is involved in the acclimation response in ETI under acute UV-B.

## 4. Materials and Methods

### 4.1. Microalgal Strains and Growth Conditions

Four microalgal strains were used in this work (Table 2). The *Chlorella sorokiniana* strains F4 and LG1 are part of the collection of the Institute of Agricultural Biology and Biotechnology of the Italian National Research Council located in Pisa and were previously described [21,22]. The strains PEC (related to *Pectinodesmus pectinatus*) and ETI (related to *Ettlia pseudoalveolaris*) were isolated from the Yahuarcocha lake (Ecuador) and then characterized as described below.

The microalgal strains PEC and ETI were isolated from samples taken in the Yahuarcocha Lake, located in the Province of Imbabura, Ecuador. For the collection of the samples, a 20 µm phytoplankton mesh was used. The sampling point was recorded at 822,290 N: 10,040,688 W with a GPS. For the isolation and cultivation of the strains, Murashige and Skoog medium (4.4 g L^−1^) was used with an extra source of nitrate (5.0 g L^−1^ KNO_3_) and phosphate (0.1 g L^−1^ KH_2_PO_4_), and the pH of the medium was adjusted to 7 [49]. Before isolation, an enrichment phase was established using 150 mL flasks with 100 mL of culture media and 10% of inoculum (Yahuarcocha freshwater). The flasks were incubated at 27 ± 1 °C with a 24/0 light/dark photoperiod and a light intensity of 104.8 µmol m^−2^ s^−1^ for two weeks. The isolation and purification of the strains were performed using the streak plate and serial dilution technique [50].

For this experiment, all microalgae were grown in the laboratories of the Italian National Research Council (Pisa) in sterile tris-acetate-phosphate (TAP) medium in a growth chamber under controlled temperature (23 ± 1 °C), 16/08 h light/dark cycle, and 70 μmol m^−2^ s^−1^ PAR. Unless otherwise noted, all chemicals and reagents used in this study were purchased from Fluke-Sigma-Aldrich, Inc. (St. Louis, MO, USA).

### 4.2. Characterization of PEC and ETI Strains

Isolate microalgae strains were identified by polymerase chain reaction (PCR) and sequencing of the 23S rRNA gene. The genomic DNA was extracted using the PureLink^®^ Plant Total DNA Purification Kit (Invitrogen, CA, USA), and the PCR amplification was performed using Master Mix PCR Taq with dye (ABM, BC, Canada). A DNA product of 400 pb was generated using the forward primer p23SrV-f1 (5′-GGA CAG AAA GAC CCT ATG AA-3′) and reverse primer p23Sr-r1 (5′-TCA GCC TGT TAT CCC TAG AG-3′) [51] with an annealing temperature of 58 °C. The PCR products were sequenced by Sanger sequencing, the sequences were curated manually, and the genetic identification was performed through the NCBI BLAST. The isolated strains are presented at the species level, and the sequences were submitted to the GenBank.

### 4.3. UV-B Radiation Treatment

A volume of 10 mL of microalgal culture was added to Petri dishes (60 × 15 mm, Greiner Bio-one, Kremsmünster, Austria). UV-B radiation was applied from a Philips TL 20W/01RS UV-B Narrowband lamp (Koninklijke Philips Electronics, Eindhoven, The Netherlands) with a peak emission at 311 nm. The intensity of UV-B was determined by adjusting the distance between the UV-B lamp and Petri dishes and measured using a UV-B meter (Skye Instruments Ltd., Powys, UK). The UV-B exposure level was set at 1.7 W m^−2^ supplemented with continuous PAR. A parallel UV-B untreated microalgal group, which only received PAR, was used as control. Culture conditions were maintained at a controlled temperature of 23 ± 1 °C and 70 μmol m^−2^ s^−1^ PAR. The experiment lasted 18 h, and microalgal cultures were collected at specific time points (0, 6, 12, and 18 h). Samples of UV-B-treated and -untreated microalgae were centrifuged at 3000× *g* for 10 min, and the microalgal pellets were collected for further analysis.

### 4.4. Extraction and Determination of Photosynthetic Pigments

Pigments were extracted from microalgal pellets in acetone 80% (*v*/*v*) and analyzed as previously reported [52]. The absorbance of extracts was spectrophotometrically measured at 470.0, 663.2, and 646.8 nm. The concentration of chlorophyll *a* (Chl*a*), chlorophyll *b* (Chl*b*), and total carotenoids (Car) were calculated by the equations of Lichtenthaler [53]. Three biological replicates were considered for these analyses.

### 4.5. Extraction and Determination of Total Antioxidant Capacity and Phenolic Compounds

Microalgal pellets were extracted in ethanol 80% (*v*/*v*) as described in Huarancca Reyes et al. [31]. Briefly, samples were sonicated for 30 min, incubated for 30 min in the dark, and then centrifuged at 10,000 rpm for 10 min. The ethanolic extracts were recovered and used for determining the total antioxidant capacity (TAC) and phenolic compounds. TAC was spectrophotometrically determined at 515 nm by the 2,2-diphenyl-1-picrylhydrazyl (DPPH) assay, as reported [54]. Phenolic compounds were spectrophotometrically determined at 750 nm with the method based on Folin–Ciocalteu reagent as described [55]. Three biological replicates were considered for these analyses.

### 4.6. Chlorophyll a Fluorescence

Chlorophyll *a* fluorescence was measured in control and UV-B treated microalgae using a portable pulse-amplitude-modulated fluorometer (Mini-PAM; Heinz Walz GmbH, Effeltrich, Germany) as previously described [56] with some modifications for algal suspensions. Briefly, the fluorescence probe was positioned with a constant distance and angle on the surface of each microalgal culture just after 18 h of treatment. The photon yield of photosystem II (PSII) photochemistry in the light (Φ_PSII_) was determined at the growth PAR of 70 μmol m^−2^ s^−1^. The potential efficiency of PSII photochemistry (*F_v_*/*F_m_*) was determined in 30 min pre-darkened microalgal cultures. All measurements included six biological replicates.

### 4.7. Statistical Analyses

Each experiment was repeated at least three times. All values shown in bar graphs are mean ± standard deviation (SD). The statistical comparison between the groups was assessed using Student’s *t*-test with statistical significance of the difference with *p* < 0.05 marked with asterisks. The software STATISTICA for Windows version 14.0 (Stat-Soft, Inc., Tulsa, OK, USA) was used.

To identify relationships among the different UV-B treated microalgal strains at the end of the experiment, based on the physiological data, multiple factor analysis (MFA) was carried out as described in Chiellini et al. [21]. The R software packages “FactoMineR” (analysis) and “factoextra” (visualization) were applied (R version 4.2.1; https://www.r-project.org/).

## 5. Conclusions

This study demonstrated the differential effects of UV-B on the inhibition or activation of protective mechanisms (i.e., production of photosynthetic pigments and antioxidants) to maintain cells photosynthetically active and how this was related to the natural environment of microalgal strains and their genetic constitutions; thus, highlighting the importance of evolutionary and ecological constraints in the final microalgal phenotype [57,58,59,60]. In detail, UV-B significantly compromised all the physiological parameters in F4, thereby resulting in the most UV-B-sensitive strain. Contrarily, UV-B exposure did not lead to changes in the PEC physiological traits, which may reflect the acclimation strategy to its natural ecosystem, maintaining a constitutive phenotype at the photosynthetic level and, thus, resulting in the strain being best resistant to acute UV-B. Differently, the prolonged UV-B exposure of LG1 and ETI resulted in species-specific metabolic regulation (e.g., non-enzymatic antioxidants) in order to constrain full photoinhibition under acute UV-B.

## Figures and Tables

**Table 1:** List of microalgal strains sequenced in this study.

Strain	Division	GenBank Name	GenBank Accession Number	Identity	Reference
PEC	Chlorophyta	*Pectinodesmus pectinatus* (KU847995.1)	OP136070	99%	[24]
ETI	Chlorophyta	*Ettlia pseudoalveolaris* (LR215808.1)	OP136137	100%	[25]

**Table 2:** List of microalgal strains.

Strain	Isolation Source	Taxonomic Affiliation	Accession Number	Reference
F4	“Le Morette”, Fucecchio Marshland	*Chlorella sorokiniana*	OM311005 and OM311000	[22]
LG1	Recycled cigarette butts substrate	*Chlorella sorokiniana*	ON065975	[21]
PEC	Yahuarcocha Lake	*Pectinodesmus pectinatus*	OP136070	This work
ETI	Yahuarcocha Lake	*Ettlia* *pseudoalveolaris*	OP136137	This work

## Data Availability

Data are contained within the article.
